# Case Report and Literature Review of Insulinoma in the Geriatric Population: An 86-Year-Old Female with Syncope of Unknown Origin

**DOI:** 10.1155/2020/8879776

**Published:** 2020-07-17

**Authors:** Gerry Samantha Eichelberger, Jordan Carbono, Zachary Field, KanwarAnoop Kainaur, Federico Montalvo

**Affiliations:** ^1^Florida State University College of Medicine, 1115 W. Call Street, Tallahassee, FL, USA 32304; ^2^Department of Internal Medicine, Orlando Regional Healthcare, 21 W. Columbia St., Orlando, FL, USA 32806; ^3^Department of Pathology, Orlando Regional Healthcare, 52 W. Underwood St., Orlando, FL, USA 32806

## Abstract

Insulinomas are extremely rare pancreatic endocrine tumors. The tumor is characterized by endogenous hypersecretion of insulin and ensuing development of symptoms of neuroglycopenia and the catecholaminergic response. Symptoms may not always be present, particularly in patients compensating appropriately with increased appetites and caloric intake due to low glucose levels. Early localization of the disease is essential to prevent lethal hypoglycemia and timely treatment. This case report and literature review depict the case of a pancreatic insulinoma in an 86-year-old female, an exceptionally rare presentation based on age and absence of clinical symptoms for one or more years prior to hospitalization. Despite its rarity, similar presentations have been reported in the literature and are further outlined with characteristics and treatment plans. This case highlights a unique presentation of insulinoma and suggests the need for clinical vigilance and further study. It also discusses diagnosis, localization, and management of this uncommon disease in patients above the age of seventy-five.

## 1. Introduction

Pancreatic endocrine tumors are unusual findings. Among pancreatic endocrine tumors, the most common type is an insulinoma. Insulinomas are in fact quite rare with an annual incidence of four cases per million people in the general population [1]. Insulinomas occur more frequently in women than in men, with a 3 : 2 female-to-male ratio [2]. No racial predilection appears to exist. These tumors typically present in the fifth decade of life with a median age of diagnosis of 47 years of age, except in patients with a history of multiple endocrine neoplasia (MEN) type one syndrome, in whom the median age is typical during the second decade of life [3]. Insulinomas are typically sporadic, benign tumors, with a solitary small (<2 cm in diameter) mass [4]. However, 10% of insulinomas are multiple and occur as a part of MEN type one syndrome. The diagnosis is often delayed or missed due to the rarity of insulinomas and nonspecific symptoms.

Most symptomatic patients present with hypoglycemic episodes resulting from inappropriate intermittent insulin secretion by the tumor. Symptoms can include diaphoresis, tremor, palpitations, tachycardia, and neuroglycopenic symptoms such as personality changes, visual disturbances, weakness, confusion, syncope, seizures, and even coma. Diagnosis is achieved by satisfying the criteria of Whipple's triad—hypoglycemia (plasma glucose <50 mg/dL), neuroglycopenic symptoms, and prompt relief of symptoms following the administration of glucose. The gold standard for biochemical diagnosis includes measuring plasma glucose, insulin, C-peptide, and proinsulin during a 72-hour fast. This prolonged fasting test can detect up to 99% of insulinomas. Approximately 65% of patients will experience hypoglycemic episodes within the first 24 hours of fast [5].

Additionally, more than one imaging modality may be necessary for the localization of the tumor. Localization is performed using computed tomography (CT), magnetic resonance imaging (MRI), endoscopic ultrasonography (EUS), intra-arterial calcium stimulation test with hepatic venous sampling or angiography, and arterial stimulation venous sampling (ARVS). Because of their small size, localization is often difficult. Diagnosis of this pathology relies on a high index of suspicion based on clinical findings and symptomatology along with laboratory testing and imaging to aid in localization. Although medical therapy can be used in the initial treatment, surgical removal is often curative and is the standard of care to prevent permanent neurological deficits associated with prolonged hypoglycemia [6]. Nonetheless, surgery in the elderly population, particularly above the age of 75, can present with a greater risk of complications.

## 2. Case Presentation

An 86-year-old Caucasian female presents to the emergency room after a syncopal episode. She gives a history of multiple syncopal episodes in the past. Her most recent episode was three months ago and resulted in a fracture of her right femur, which was subsequently treated surgically. Detailed cardiac and neurological evaluations at that time were unable to establish a clear etiology for her syncope. The rest of her pertinent past medical history includes essential hypertension, hyperlipidemia, and breast cancer for which she underwent a bilateral mastectomy. Emergency responders found the patient unresponsive with a blood glucose of 38 mg/dL (70–99 mg/dL fasting). The patient received 50 mL of 50% dextrose in water and became responsive. Repeat glucose was 260 mg/dL. Upon further questioning, the patient admitted to skipping lunch to explain her low blood sugar. She also admitted to having multiple falls of unclear etiology during the past year.

At the time of her current admission, vital signs were notable for an elevated blood pressure of 151/70 mmHg. Physical examination, including complete neurological examination, was normal. Postural blood pressure readings did not show any orthostatic hypotension. Basic laboratory studies including a complete metabolic panel and complete blood count were normal except for the glucose level. Glucose was noted to be 125 mg/dL (70–99 mg/dL fasting). Her hemoglobin A_1_C was noted to be 4.7% (4–5.6%); C-peptide was noted to be 2.16 ng/mL (0.78–5.19 ng/mL). Additional workup including TSH, electrocardiogram, troponins, and computerized tomography of the head were within normal limits.

Throughout the hospital course, the patient experienced recurrent hypoglycemic episodes. Fasting blood sugar was as low as 56 mg/dL on day three of her hospitalization requiring frequent doses of intravenous dextrose. Eventually, a continuous dextrose infusion was needed to maintain adequate blood glucose levels. The patient was tested for adrenal insufficiency with a morning cortisol level, which was found to be normal. Additionally, she was screened for exogenous hypoglycemic agents including sulfonylurea levels, which were negative.

Finally, the patient underwent a 72-hour fasting trial to assess for an insulinoma. The patient experienced symptomatic hypoglycemia within 48 hours, with a blood glucose of 32 mg/dL. Fasting trial was discontinued and insulin and C-peptide levels were 22.05 ulU/mL (1.90–23.00 ulU/mL) and 3.61 ng/mL (0.78–5.19 ng/mL), respectively, during the time of the hypoglycemic episode. High normal insulin and normal C-peptide levels in conjunction with very low plasma glucose levels strongly indicated the presence of an insulinoma.

**Figure 1:**
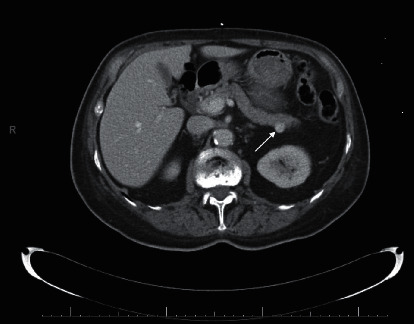
Computerized tomography of the abdomen and pelvis demonstrating enhanced pancreatic tail mass (1.5 cm) suggestive of insulinoma in the setting of unexplained hypoglycemia.

**Figure 2:**
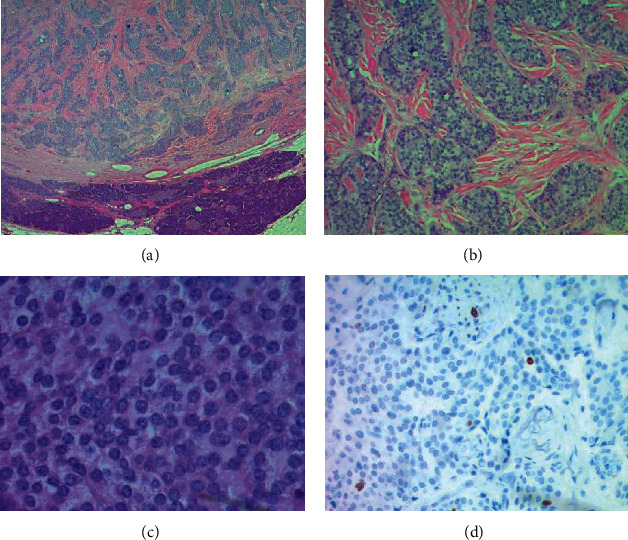
Low-grade pancreatic neuroendocrine tumor. (a) Low-power (20x) view shows a well-circumscribed nested pattern of the tumor cells within the eosinophilic stromal background with adjacent pancreatic parenchyma and (b) 100x view. (c) 400x exhibits uniform nuclei with “salt and pepper” chromatin. (d) 200x shows an immunohistochemical stain for Ki67, which demonstrated 1% reactivity, supporting a low-grade designation.

Shown in Figure 1, the computerized tomography scans of the chest, abdomen, and pelvis demonstrated a well-defined enhancing pancreatic tail mass highly suggestive of insulinoma. The computerized tomography scans ruled out metastatic disease. The patient underwent elective laparoscopic-assisted pancreatic mass enucleation on day twelve of her hospitalization. Pathology results showed a low-grade well-differentiated noninvasive pancreatic neuroendocrine tumor, 1.5 cm diameter in size with no invasion present and negative margins as depicted in Figure 2.

Postoperatively, blood glucose levels normalized, without requiring any additional dextrose. The patient was discharged three days later. Two months after her hospitalization, the patient continued to show improvement in her clinical symptoms. She did not report any additional syncopal episodes or falls. Follow-up laboratory work demonstrated euglycemia. Repeat insulin and C-peptide levels were not obtained because of symptomatic improvement.

## 3. Discussion

Insulinomas have variable and nonspecific presentations particularly in the elderly population usually related to the hypoglycemic episodes. This can be attributable to the episodic and intermittent nature of the secretion of insulin from the tumor. Moreover, the pathogenesis of insulinomas remains unclear. In patients with insulinomas, there is continued secretion of insulin despite a low glucose level. Catecholaminergic and neuroglucopenic symptoms aid in reaching the diagnosis. However, due to the wide range of symptoms and severity, insulinomas can go undiagnosed for a substantial length of time. A broad differential diagnosis of syncope in the geriatric population further prevents prompt diagnosis of insulinoma. In fact, the median duration of symptoms before diagnosis remains variable and can be 12–18 months on average or even years in rarer circumstances from the onset of symptoms [7]. Delayed diagnosis and treatment of insulinomas represents a potentially dangerous condition. Early diagnosis prevents cognitive impairment as well as falls and improves the quality of life of elderly patients.

Insulinomas are defined by meeting Whipple's triad including hypoglycemia (plasma glucose <50 mg/dL), neuroglycopenic symptoms, and relief of symptoms following the administration of glucose. The 72-hour fasting test remains the gold standard for biochemical diagnosis with measurements of plasma glucose, insulin, C-peptide, and proinsulin levels during the onset of the hypoglycemic episodes. Various imaging modalities may be used to localize the tumor. Imaging type may be limited to what is available at each institution. However, studies have shown a higher sensitivity and preference for helical computerized tomography and endoscopic ultrasound, both of which have up to 94% sensitivity [8]. For most insulinomas, surgery can be curative. Alternatively, medical treatment with octreotide or streptozotocin may be used especially in those with contraindications to surgery or metastatic disease. In cases of malignant insulinomas, an aggressive medical approach is used for enhancing the quality of life by preventing hypoglycemic episodes and improving survival.

Exposure and recognition of an insulinoma case may be limited due to lack of clinical experience amongst healthcare providers. This is especially true in the event of an atypical presentation such as diagnosing insulinomas in the geriatric population. The importance of this case outlines the difficulty in diagnosing insulinomas in patients above the age of seventy-five. Syncopal episodes increase during aging with a prevalence of more than 20% in patients above the age of 75 [9]. Multiple etiologies of syncope exist including cardiovascular disease, arrhythmias, orthostatic hypotension, and neurological causes [10]. Risk factors for hypoglycemia in geriatric patients can include baseline dementia, polypharmacy, and inadequate diet, all of which are common characteristics of patients within this age group, making a diagnosis of an insulinoma more challenging [11]. Nonetheless, it is critical for healthcare providers to maintain insulinoma as a potential diagnosis in this patient population. Missing the diagnosis can lead to morbidity and mortality.

Despite the rarity of this disease, clinicians should be aware of the occurrence of insulinomas in the geriatric population, and how the clinical manifestations can widely vary.  Table 1 depicts insulinoma cases in the geriatric population reported in the literature. Insulinomas are not restricted to a particular demographic or age group, as demonstrated by this case. While the classic demographic for insulinomas presents between 40 and 60 years of age, this patient presented at 86 years old, illustrating the wide demographic variance. Creating a proper differential diagnosis and having a high clinical index of suspicion will aid in making a diagnosis earlier. Healthcare providers that encounter syncope in the setting of hypoglycemic episodes should still consider the diagnosis of insulinoma in a geriatric patient. Ultimately, the best treatment is surgical removal or enucleation [11].Laparoscopic surgery can be performed; however, in approximately 44% of cases, open exploration was needed presenting greater possibility of complications [17]. Thus, in the geriatric population, particularly in those above the age of 75, the treatment plan can be medical or surgical. Due to the higher risks of surgical and postsurgical complications in this population, patients and physicians should engage in shared medical decision-making. However, with advancements in surgical techniques, more data are necessary to assess the relative risk and mortality from surgical management.

## 4. Conclusion

Insulinomas are a rare pancreatic endocrine tumor and disease, but can cause severe neurologic damage and complications resulting from repeated hypoglycemia in geriatric patients. Insulinomas are a difficult diagnosis to make and require a high index of clinical suspicion focusing on the significant recurrent hypoglycemic episodes. It is imperative for physicians to keep in mind insulinomas as a rare cause of syncope in patients above the age of 75 with hypoglycemic episodes, as missing the diagnosis can ultimately lead to morbidity and mortality. Patients and physicians should engage in shared medical decision-making of whether to opt for surgical or medical management, especially in the geriatric population who may be particularly vulnerable to complications.

## Figures and Tables

**Table 1:** Outline of the literature with insulinoma cases including characteristics of patients, image modality of choice for diagnosis, location of tumor, and treatment plan.

Literature review of insulinomas in elderly patients (above 75 years of age)												
Name of study	Year	Age of presentation	Male vs. fatale	History of multiple endocrine neoplasia	Initial glucose level (mg/dL)	Glucose level within 72-hour fast trial	Imaging modality for diagnosis	Tumor size and location	Metastasis	Surgical management	Medical management

An elderly patient with an insulinoma who had prolonged dementia-like symptoms [12]	1998	77	Female	—	34 mg/dL	—	Abdominal ultrasound; mass not seen on CT or MRI; tumor localized via abdominal arteriography	1 cm tumor localized at the pancreatic tail	—	Pancreatectomy	—

Malignant insulinoma: spectrum of unusual clinical features [13]	2005	82	Female	No	26 mg/dL	—	Abdominal MRI	8.0 cm tumor localized at the dome of the liver	Yes	Percutaneous radiofrequency ablation	—

Successful treatment of insulinoma by a single daily dose of octreotide in two elderly female patients [14]	2006	76	Female	No	—	39 mg/dL after 6 hours	Abdominal angiography	1.5 cm tumor localized at the dome of the liver	No	Refused surgical management	Octreotide 50 *μ*g subcutaneous once daily

Successful treatment of insulinoma by a single daily dose of octreotide in two elderly female patients [14]	2006	85	Female	No	61 mg/dL	37 mg/dL	Abdominal CT	1.5 cm tumor localized at the pancreatic uncus	No	Unsuitable for surgery due to obesity	Initial management was octreotide 100 *μ*g subcutaneous once daily and subsequently reduced to octreotide 50 *μ*g subcutaneous once daily

Pancreatic insulinoma coexisting with gastric GIST in the absence of neurofibromatosis 1 [15]	2009	76	Female	No	—	—	Abdominal CT	1.3 cm localized at the pancreatic tail	No	Pancreatectomy	Initial management with diazoxide

Insulinoma in the elderly: a report of 3 cases and review literature [11]	2014	82	Female	Yes	—	—	Abdominal MRI	Unknown size; location initially pancreas with metastasis to liver	Yes	Pancreatectomy w/partial hepatectomy (1993); radiofrequency (2004 and 2006); selective angiographic embolization (2010)	Metastasis in 2011: initial management with subcutaneous injection of octreotide 0.1 mg twice a day and switched to long-acting lanreotide 120 mg every four weeks upon discharge; further progression of metastasis in 2012; lanreotide was switched to diazoxide 100 mg three times a day w/symptomatic improvement

Insulinoma in the elderly: a report of 3 cases and review literature [11]	2014	84	Male	No	—	—	Abdominal MRI	1 cm tumor localized at pancreatic tail that progressed to 2.2 cm in size	No	Refused surgery (2006); received unsuccessful selective embolization (2012)	Octreotide 0.2 mg three times a day

Insulinoma in the elderly: a report of 3 cases and review literature [11]	2014	85	Male	No	—	—	Abdominal MRI	4 cm tumor localized at the pancreatic head	No	Refused surgical management	Initial management with subcutaneous injection of octreotide 0.1 mg twice a day and switched to long-acting lanreotide 120 mg every four weeks upon discharge

Octreotide LAR was useful for avoiding hypoglycemia in an elderly patient with insulinoma [16]	2017	80	Female	No	—	—	—	—	No	Refused surgical management	Initial management with octreotide 50 mg subcutaneous injection every day; subsequently switched to lanreotide 120 mg every 4 weeks

Insulinoma case report and literature review of insulinoma in 86-year-old female with late onset hypoglycemia of unknown origin	2020	86	Female	No	38 mg/dL	32 mg/dL	Abdominal CT	1.6 cm tumor localized at pancreatic tail	No	Pancreatectomy with enucleation	—

## Data Availability

All relevant data used to support the findings of this study are included within the article. Previously reported literature review data were used to support this study. These prior studies (and datasets) are cited at relevant places within the text as references [11–16].
